# Mini-Dose Glucagon for Mild and Moderate Hypoglycemia in Type 1 Diabetes

**DOI:** 10.18103/mra.v12i10.5866

**Published:** 2024-10-29

**Authors:** Hal Steven Farkas, Ellen Werber Leschek

**Affiliations:** 1 College of Osteopathic Medicine, Nova Southeastern University, Fort Lauderdale, FL; 2 National Institute of Diabetes and Digestive and Kidney Diseases, National Institutes of Health, Bethesda, MD 20892

## Abstract

Tight glycemic control reduces the development and progression of microvascular and macrovascular complications in individuals with type 1 diabetes. However, it is also associated with an increased risk of hypoglycemia, which can deter some individuals from striving to achieve tight glycemic control. For this and other reasons, it is important to optimize the treatment of hypoglycemia in type 1 diabetes. The conventional approach to the management of mild to moderate hypoglycemia is oral carbohydrate ingestion, which can result in significant rebound hyperglycemia and the consumption of excess calories with resulting unwanted weight gain. Studies of the use of subcutaneous mini-dose glucagon for the prevention and treatment of mild to moderate hypoglycemia in a variety of settings have demonstrated that this approach is safe and effective and is less likely to result in significant hyperglycemia and the ingestion of unwanted oral carbohydrate calories.

## Introduction

Hyperglycemia is a direct cause of the development and progression of microvascular and macrovascular complications in individuals with type 1 diabetes (T1D).^1^ For this reason, T1D intervention is aimed at the achievement of glycemia as near to the non-diabetic range as possible. However, tight glycemic control is associated with an increase in the risk of hypoglycemia.^2,3^ The risk of hypoglycemia in people with T1D has lessened with the advent of closed-loop hybrid insulin delivery systems, which aims to reduce both hyperglycemia and hypoglycemia.^4^ However, the risk still exists, and both hypoglycemia and fear of hypoglycemia can be obstacles to the achievement of optimal glycemic control in individuals with T1D.^5^

International Society for Pediatric and Adolescent Diabetes (ISPAD) guidelines define hypoglycemic events as blood glucose concentrations low enough to cause signs and/or symptoms. Glucose values of <3.9 mmol/L (<70 mg/dL) are the threshold for clinical alert and initiation of treatment because of the potential for glucose level to fall further. Glucose levels of <3.0 mmol/L (<54 mg/dL) are serious, as they can cause neurologic symptoms, including cognitive dysfunction. Factors that contribute to hypoglycemia in T1D include excess insulin, decreased food consumption, exercise, sleep and alcohol ingestion.^5^

The outpatient treatment of severe hypoglycemia in T1D, involving loss of consciousness and/or the inability to swallow, principally involves the administration of intramuscular, subcutaneous or intranasal glucagon. The treatment of individuals with T1D and mild to moderate hypoglycemia (those who are conscious and able to swallow) starts with oral administration of 0.3 mg/kg of a fast-acting carbohydrate. Often glucose tablets are used for this purpose, but if they are not available, fruit juice, regular soft drink, honey or sugary candy may be used. Blood glucose is then rechecked in 15 minutes and if the individual is still hypoglycemic, administration of fast acting carbohydrate is repeated. This cycle is continued until glucose is >3.9 mmol/L (>70 mg/dL), at which time a 10–15 g oral carbohydrate snack is given.^5^

The treatment of mild to moderate hypoglycemia can result in significant hyperglycemia as well as the consumption of excess calories, which can lead to unwanted weight gain.^6,7^ Studies of subcutaneous mini-dose glucagon (MDG) administration for mild to moderate hypoglycemia (as well as for hypoglycemia prevention) in individuals with T1D have demonstrated that this approach may help to avoid some of the unwanted effects of fast-acting oral carbohydrate.

## Literature Review

Mini-dose glucagon can be an important tool for treating and preventing mild to moderate hypoglycemia in individuals with T1D. This approach may be more efficient and accurate than fast-acting oral carbohydrate and may help to avoid some of its unwanted effects, including hyperglycemia and weight gain. Mini-dose glucagon has been tested in a variety of settings.

### POTENTIAL SETTINGS FOR MINI-DOSE GLUCAGON USE FOR MILD TO MODERATE HYPOGLYCEMIA

#### A. Gastroenteritis and/or Poor Oral Intake

In 2001, Haymond and Schreiner published a manuscript evaluating MDG as a strategy for addressing mild or impending hypoglycemia in children with T1D and gastroenteritis and/or poor oral intake.^8^ Mini-dose glucagon was administered by parents at home to 28 children with impending or mild hypoglycemia when blood glucose was <4.44 mmol/L (<80 mg/dL) by home meter. Children ≤2 years of age were given 20 μg subcutaneously and children >2 years were given 150 μg. If blood glucose did not increase to >4.44 mmol/L (>80 mg/dL) within 30 minutes, treatment was repeated with a double dose of MDG. Fourteen children required a second MDG treatment due to recurrence of hypoglycemia, and four required a third. Thirty-three hypoglycemic episodes were analyzed in the 28 children. Blood glucose was 3.44 ± 0.15 mmol/L (62.0 ± 2.7 mg/dL) before MDG treatment and 8.11 ± 0.72 mmol/L (146.1 ± 13.0 mg/dL) 30 minutes after treatment. Mini- dose glucagon did not cause or worsen nausea or emesis, and none of the MDG-treated children required hospitalization due to hypoglycemia. The investigators concluded that MDG rescue using this treatment regimen was effective in the management of T1D children during periods of impending hypoglycemia due to gastroenteritis and/or poor carbohydrate intake.

Five years later, Hartley et al published results of an assessment of subcutaneous MDG use in children with T1D and mild or impending hypoglycemia associated with inability or refusal to take oral carbohydrate.^9^ Children with blood glucose level <4.0 mmol/L (<72 mg/dL) were given MDG 20 μg subcutaneously if they were ≤2 years of age, and children >2 years were given an additional 10 μg per year of age up to a maximum of 150 μg. Blood glucose was rechecked by home meter after 20 minutes, and if it was still <4.0 mmol/L (<72 mg/dL), treatment was repeated with a double dose of MDG. Over two years, 25 children received MDG according to this protocol on a total of 38 occasions, and 20 additional doses were administered for hypoglycemia recurrence. Of the 38 episodes of hypoglycemia treated with MDG, only one child was admitted to the hospital for persistent hypoglycemia. This child received three MDG injections over 75 minutes, but the dose had not been doubled after the initial suboptimal response per instructions. Hartley et al found that they were successfully able to reproduce Haymond and Schreiner’s treatment regimen and results, concluding that this was an effective management approach for children with T1D and mild or impending hypoglycemia due to gastroenteritis and/or food refusal.

In 2010, Busselo et al published the outcome of a retrospective evaluation of the efficacy and safety of MDG administration in the Emergency Department for impending or mild hypoglycemia due to acute gastroenteritis and/or persistent vomiting in children with T1D.^10^ They analyzed 7 children who received MDG and 21 children who did not receive MDG between 2004 and 2010. Those who received MDG had an average pretreatment blood glucose of 4.5 mmol/L (82 mg/dL) (range 3.6–6.3 mmol/L [64–114 mg/dL]) and those who did not receive MDG had an average pretreatment blood glucose of 5.2 mmol/L (93 mg/dL) (range 2.3–6.9 mmol/L [42–124 mg/dL]). Of the 7 children who received MDG, only 1 (14.3%) required hospital admission and 1 (14.3%) required intravenous fluid therapy. Of the 21 who did not receive MDG, 16 (76.2%) required hospital admission and 18 (85.7%) needed treatment with intravenous fluid. The authors concluded that MDG was very effective in managing hypoglycemia and preventing the need for intravenous fluid administration and hospitalization in children with T1D and gastroenteritis and/or persistent vomiting.

In 2016, Chung and Haymond reviewed previously published data on the use of MDG for mild or impending hypoglycemia with gastroenteritis and/or inability/refusal to consume oral carbohydrate.^11^ They pointed out that MDG was being widely utilized off-label for the management of mild or impending hypoglycemia to prevent severe hypoglycemic episodes. They emphasized its safety, efficacy and ease of administration. The review included the MDG algorithm they were using to manage T1D children and adolescents in their practice who had mild or impending hypoglycemia with gastroenteritis and/or refusal to eat, which was consistent with the treatment approach described in their 2001 publication. They also noted that regimens comparable to this had been implemented by multiple children’s hospitals and organizations across the world. They emphasized that at that time, glucagon was only available as a 1 mg emergency kit containing crystallized powder requiring reconstitution prior to use. Once reconstituted, the glucagon peptide, an aqueous form, dimerized and degraded quickly and thus had to be discarded within 24 hours. The kit was costly, adding the potential for significant financial burden for this off- label glucagon use. However, they did note that prevention of severe hypoglycemia and visits to the emergency department would likely more than compensate for the cost of the kits. At the time of this review, alternate, more stable forms of glucagon (injectable and nasal) were under development but not yet available.

Tinti et al published data in 2019 from a case series involving the use of MDG in 3 children with T1D and impending or mild hypoglycemia (4 episodes) who refused oral carbohydrate.^12^ Mini-dose glucagon was given at a dose of 10 μg subcutaneously for every year of age up to a maximum of 150 μg. The same dosage was repeated in 30 minutes if blood glucose did not increase. Continuous Glucose Monitor (CGM) data showed that following MDG injection, blood glucose was 7.0 ± 4.4 mmol/L (127 ± 80 mg/dL) after 1 hour and 9.2 ± 4.3 mmol/L (165 ± 78 mg/dL) after 2 hours. There was only one recurrence of hypoglycemia requiring a second dose of MDG. None of the children required hospitalization due to hypoglycemia. The investigators concluded that MDG was both safe and effective in this setting.

#### B. Exercise

The first study evaluating MDG as a novel treatment to prevent exercise-induced hypoglycemia was published in 2018.^13^ This study, performed by the T1D Exchange Mini- Dose Glucagon Exercise Study Group, compared subcutaneous MDG administration before exercise to current approaches for prevention of hypoglycemia in this setting. Fifteen adults with T1D for ≥2 years were enrolled in a randomized crossover trial. Each completed 4 exercise sessions within a 12-week period. The 45-minute exercise sessions involved brisk walking on an inclined treadmill. There was no intervention during the control session, basal insulin reduction by 50% during one session, administration of oral glucose tablets (20 g at start and 20 g 30 minutes into exercise) during one session and MDG (150 μg) administration during one session. All interventions were delivered 5 minutes before the start of exercise. The study showed that glucose decreased during the control and basal insulin reduction sessions and increased slightly with MDG. Thirty minutes into exercise, glucose increased more when oral glucose tablets were used compared to MDG (p < 0.001). Six participants experienced hypoglycemia (glucose <3.9 mmol/L [<70 mg/dL]) during the control arm, five during the reduced basal insulin infusion rate arm and none during the oral glucose and MDG arms. Five participants experienced hyperglycemia (glucose ≥13.9 mmol/L [≥250 mg/dL]) during the oral glucose arm and one during the MDG arm. The study group concluded that MDG appeared to be more effective than a 50% reduction in insulin basal rate for the prevention of exercise-induced hypoglycemia and may cause less post- exercise hyperglycemia than oral glucose.

Results from a randomized, placebo-controlled, participant-blinded study looking at glucose increase after MDG in adults with longstanding T1D were published by Steineck et al in 2019.^14^ Fourteen insulin-pump-treated individuals completed three study visits. At each visit, they consumed a standardized breakfast 2 hours before 45 minutes of either cycling or resting. Mini-dose glucagon 200 μg was given subcutaneously before or after cycling or resting. Glucose response (change) was higher after cycling compared to resting (2.6 ± 1.7 versus 1.8 ± 2.0 mmol/L [46.8 ± 30.6 versus 32.4 ± 36.0 mg/dL], p = 0.02). Glucose decreased during cycling, as expected (−3.1 ± 2.8 mmol/L [-55.8 ± 50.4 mg/dL], but the decrease was less when glucagon was administered prior to cycling (−0.9 ± 2.8 mmol/L [-16.2 ± 50.4 mg/dL], p = 0.002). The investigators concluded that cycling did not attenuate the glucose response to glucagon (compared to resting) and that the glucose fall during cycling was diminished by pre-exercise MDG treatment.

The most recent study of MDG, evaluating its use in the prevention of exercise-associated hypoglycemia in adults with T1D on continuous subcutaneous insulin infusion (CSII) pumps, was published by Aronson et al in 2023.^15^ The investigators formulated ready-to-use (room temperature-stable) MDG (150 μg in 30 μL) and matching placebo (30 μL) vials. Forty-five participants were randomly assigned to 12 weeks of one of the following interventions immediately before 30–75 minutes of moderate to high intensity exercise: (1) 50% reduction in basal insulin rate plus MDG 150 μg (blinded), (2) 50% reduction in basal insulin rate plus placebo (blinded), or (3) MDG 150 μg (open label) alone. Incidence of level 1 hypoglycemia (3.0–3.8 mmol/L [54–69 mg/dL]) was significantly lower in both MDG arms (12% in the blinded arm and 16% in the open label arm) compared to the placebo arm (39%).

Times below, in and above range did not differ between arms. The investigators concluded that MDG with or without reduction in insulin basal rate appeared to decrease exercise-associated hypoglycemia in adults with T1D.

#### C. Fasting

Greater than 40% of adult Muslims with T1D fast during Ramadan and do not want to invalidate their fast by using oral glucose for hypoglycemia, posing a considerable risk. In 2022, Algeffari et al published results from a 4-week randomized, controlled crossover trial of MDG in 17 adults with T1D to evaluate an alternative intervention for individuals who chose to fast for ~15 hours per day during Ramadan.^16^ All participants wore a CGM and were treated with either MDG or oral glucose tablets (control) for each episode of fasting-induced hypoglycemia over a 2-week period. Each subsequently crossed over to the alternate intervention for 2 weeks. Treatment involved administration of 150 μg MDG or 15 g oral glucose for blood glucose 2.8–3.8 mmol/L (50–69 mg/dL) and 300 μg MDG or 30 g oral glucose for blood glucose 2.2–2.7 mmol/L (40–49 mg/dL). Among the 17 participants, 80 hypoglycemic episodes occurred. Compared to oral glucose, MDG resulted in a significantly greater increase in blood glucose from baseline at both 30 and 60 minutes, and efficacy was preserved after ≥8 hours of fasting. During the 2 weeks of MDG use, time in the 3.9–10.0 mmol/L (70–180 mg/dL) range was increased. Overall, MDG use was associated with a higher rate of valid fast completion compared with oral glucose. The investigators concluded that MDG was an effective alternative for the prevention and treatment of hypoglycemia due to fasting. It improved glycemic control and allowed successful completion of prolonged fasts.

#### D. Summer Camp

A letter to the editor written by Drs. Hasan and Kabbani and published in the *Journal of Pediatrics* in 2004 described the investigators’ experience with the use of Haymond’s MDG algorithm to treat hypoglycemia in T1D children at a diabetes camp.^17^ Increased physical activity at summer camp can result in more frequent episodes of hypoglycemia, particularly in children with tight metabolic control prior to camp. During the summer prior to this publication, the authors reported 28 episodes of severe hypoglycemia (requiring the assistance of another person) among their 220 campers and 50 young adult counselors with T1D. During 21 (75%) of these, the individual was alert enough to be treated with oral glucose. Of the other 7, 2 had seizures and required full dose (1 mg) glucagon in addition to intravenous glucose (dextrose) treatment. The other 5 experienced confusion and inability to consume sufficient oral glucose but no loss of consciousness. These 5 campers were treated with MDG according to the Haymond protocol.^8^ All 5 had blood glucose <3.3 mmol/L (<60 mg/dL) (average 2.3 mmol/L ± 0.5 mmol/L [42.3 ± 9.2 mg/dL]) and oral glucose had failed to correct their low blood glucose or their signs and symptoms. However, MDG resulted in near immediate recovery with blood glucose rising to >5.5 mmol/L (>100 mg/dL) (8.4 ± 1.0 mmol/L [152 ± 18.3 mg/dL]) by 15 minutes. After 30 minutes of observation, these 5 campers were able to return to their scheduled activities. No adverse events were observed due to the MDG. In summary, the investigators found MDG to be extremely effective in correcting hypoglycemia at summer camp with complete recovery of mental status and without complaints of nausea or vomiting.

#### E. Ethanol Ingestion

Ethanol intake can induce hypoglycemia in individuals with T1D about 8–12 hours after consumption. In 2018, Ranjan et al published an evaluation of the effects of MDG on hypoglycemia following ethanol intake in adults with T1D.18 This randomized, placebo-controlled, crossover trial enrolled 12 people with T1D on CSII pumps. Each participant had two overnight study visits that involved dinner at 6 PM consisting of 1 g of carbohydrate/kg of body weight and either a diet drink (placebo) or a diet drink mixed with 0.8 g/kg of ethanol. It was expected that the ethanol would be metabolized after 8–9 hours, so at 8–9 hours, a subcutaneous bolus of insulin was given to induce mild hypoglycemia. When glucose reached ≤3.9 mmol/L (≤70 mg/dL), MDG (100 μg) was given subcutaneously followed by a second dose 2 hours later. Mini-dose glucagon successfully treated the induced mild hypoglycemia following ethanol intake (glucose increased by 2.0 mmol/L [36 mg/dL]), but the effect was somewhat attenuated compared to that seen in the placebo (no ethanol) group. The investigators concluded that MDG may be a reasonably effective treatment for mild hypoglycemia following ethanol intake.

#### F. Insulin-Induced Hypoglycemia

The first dose-response study of MDG, published in 2016, evaluated a new, non-aqueous, ready-to-use glucagon formulation under development by Xeris Pharmaceuticals, Inc. Haymond et al assessed three doses of MDG (75, 150 and 300 μg).^6^ Twelve adults with longstanding T1D were treated on different days with three doses of MDG. The order of the doses was randomized. Following an overnight fast, MDG was injected in the morning and 180 minutes later, to induce hypoglycemia, participants received a subcutaneous insulin bolus intended to cover 30 g of oral glucose. Once blood glucose decreased to<3.9 mmol/L (<70 mg/dL), or 2 hours after insulin administration (whichever came first), a second identical dose of MDG was administered. Serial blood samples were obtained before and after both MDG doses. Study results revealed that the 75 μg dose of glucagon generated a suboptimal glucose response and the 300 μg dose occasionally caused mild nausea. The 150 μg dose was optimal, as it resulted in a good blood glucose response and did not cause nausea. There were some inconsistent complaints of injection site discomfort thought to be related to the dimethyl sulfoxide (DMSO) used to stabilize this new glucagon formulation. In addition to providing dose-response data for MDG use, this study also served as documentation of the safety and efficacy of this new formulation for MDG use at 150 μg, the dose previously found to be optimally effective in adults with T1D and mild to moderate hypoglycemia.

In 2017, the T1D Exchange Mini-Dose Glucagon Study Group published a paper describing their experience using the same non-aqueous MDG formulation (using DMSO for stabilization) under development by Xeris Pharmaceuticals, Inc. to treat mild hypoglycemia in ambulatory adults with T1D.^19^ Twenty individuals with T1D were enrolled in a 6-week randomized crossover trial followed by a 3-week extension phase. A 2-week run-in phase preceded randomization to assess study procedure compliance and frequency of hypoglycemia (they had to have at least one CGM value <3.9 mmol/L [<70 mg/dL] on ≥3 of the 14 days). The study consisted of 2 3-week periods. During one, the participants used MDG to treat hypoglycemia and during the other, they used oral glucose tablets to treat hypoglycemia (2.8–3.8 mmol/L [50–69 mg/dL]). During the extension phase, they could choose to use either MDG (150 μg) or glucose tablets (16 g) for hypoglycemia. Hypoglycemia throughout the study was confirmed by glucose meter. Blood glucose was rechecked 30 minutes after treatment. Sixteen participants had 118 analyzable events during the study. Treatment was considered successful for 94% of the events that were treated with MDG and 95% of the events that were treated with glucose tablets. While CGM-measured time in range was the same during the 2 hours following events, treatment with glucose tablets resulted in significantly higher maximum glucose over the first hour following treatment (6.4 versus 5.7 mmol/L [116 versus 102 mg/dL], p = 0.01). During the extension phase, ~50% of hypoglycemic events were treated with MDG and ~50% were treated with glucose tablets. Overall, this study demonstrated that this new MDG formulation could be used successfully to treat mild hypoglycemia and may be useful when trying to avoid unnecessary caloric intake.

A next-generation glucagon analog, dasiglucagon, was approved by the FDA in 2021 for the treatment of severe hypoglycemia in T1D individuals ≥6 years of age. Like other glucagon preparations, dasiglucagon was packaged as a single-dose unit.^20^ Soon after dasiglucagon approval, Laugesen et al published the results of a study comparing the efficacy of mini-dose dasiglucagon to oral glucose tablets for prevention of insulin-induced hypoglycemia in people with T1D.^21^ Twenty adults with T1D participated in a randomized, 3- arm, crossover study. At each study visit, a subcutaneous insulin bolus was given with the goal of achieving a glucose level of 3.0 mmol/L (54 mg/dL). When glucose reached 4.5 mmol/L (81 mg/dL), the participant was given either 15 g of oral glucose tablets, 80 μg of dasiglucagon or 120 μg of dasiglucagon. Hypoglycemia (<3.9mmol/L [70mg/dL]) occurred in 10 participants after oral glucose, 5 after dasiglucagon 80 μg and 4 after 120 μg dasiglucagon. Time spent in hypoglycemia (<3.9mmol/L [<70mg/dL]) was 14% for oral glucose, 7% for 80 μg dasiglucagon and 6% for 120 μg dasiglucagon. Median time from intervention to first increase in glucose of 1.1 mmol/L (20 mg/dL) was 30 minutes for oral glucose and 15 minutes for 80 μg and 120 μg dasiglucagon. The investigators concluded that dasiglucagon safely and effectively prevented insulin-induced hypoglycemia and brought blood glucose levels up significantly faster than oral glucose.

### PATIENT PREFERENCES FOR THE TREATMENT OF MILD TO MODERATE HYPOGLYCEMIA

Tezschner et al published the results of a prospective survey study in 2019 to gauge whether adults with T1D preferred treating their mild hypoglycemia with oral carbohydrate or MDG.^22^ A total of 51 participants registered all their mild hypoglycemia events, and for each, they recorded whether they would have preferred the use of MDG over oral glucose administration had MDG been available. Each participant had 10 episodes of mild hypoglycemia on average (range 3–23) for a total of 514 events over the 2-week survey period. Mini-dose glucagon was preferred in 58% and 12% of participants had no desire to use MDG. The researchers concluded that the majority of their T1D participants were interested in MDG use for the treatment of their mild hypoglycemic events.

In 2022, Hughes et al published results from a study conducted among 38 members of the T1D Exchange online community and T1D Exchange Registry.^23^ The investigators convened 6 focus groups consisting of adults with longstanding T1D to look at the emotional impact of hypoglycemia, views of glucagon and barriers to glucagon use. While most of the report focused on severe hypoglycemia and its treatment, participants indicated that they were interested in a “premixed-ready-to-go” MDG preparation that they could self-administer for less severe episodes of hypoglycemia.

### GUIDANCE FOR THE TREATMENT OF MILD TO MODERATE HYPOGLYCEMIA

In 2009, ISPAD included the use of MDG in its clinical practice consensus guidelines for sick day management of children and adolescents with T1D.24 For hypoglycemia (blood glucose <3.5–4 mmol/L [<65–70 mg/dL]) and persistence of nausea or food refusal, ISPAD endorsed the use of MDG to enable resumption of oral food intake. Specifically, they recommended MDG 20 μg subcutaneously for children ≤2 years of age, an additional 10 μg per year of age for children 2 to 15 years of age and 150 μg for children >15 years old.

In 2021, the Pediatric Endocrine Society published patient instructions for the use of MDG on their website.25 They indicate that these guidelines are meant for prevention of and early intervention during episodes of mild to moderate hypoglycemia, when blood sugar levels are <3.9 mmol/L (<70 mg/dL) and the child is awake but not able to eat or refuses to eat. They instruct patients on the preparation, storage and reuse guidelines for MDG made from a 1 mg glucagon emergency kit. Like ISPAD, they recommend 20 μg for children ≤2 years of age and the addition of 10 μg for each year of age up to a maximum of 150 μg. They advise a second, double dose of MDG if blood glucose does not rise sufficiently after 20–30 minutes.

Revised clinical practice guidelines published by ISPAD in 2022 again provide recommendations for the use of MDG for sick day management in children and adolescents with T1D.^5^ They state that children with gastrointestinal illness and/or poor oral carbohydrate intake and blood glucose ≤4.4 mmol/L (≤79 mg/dL) could benefit from injection of MDG to avoid impending hypoglycemia and hospitalization. The suggested dose is 20 μg for children ≤2 years of age and 10 μg per year of age for children ≥3 to 15 years old (maximum dose 150 μg). Repeat MDG injection at twice the initial dose is recommended if glucose fails to rise sufficiently after 30 minutes.

In 2024, Children with Diabetes published a section entitled, “Mini-Dose Glucagon Rescue for Hypoglycemia for People with Type 1 Diabetes” on their website.^26^ The page highlights the utility of MDG for the prevention and treatment of hypoglycemia in children with gastroenteritis. The authors instruct readers regarding how to draw up the proper MDG quantities from a glucagon emergency kit and how to calculate the correct dosage. The guidelines mirror those described by ISPAD and the Pediatric Endocrine Society.

## Discussion

Tight glycemic control to minimize hyperglycemia in individuals with T1D is critical for the prevention of both microvascular and macrovascular complications, including retinopathy, nephropathy, neuropathy, myocardial infarction and stroke.^1^ However, the achievement of near-normal glycemia in this population is associated with an increase in hypoglycemia risk, which can interfere with the attainment of optimal glycemic control.

Currently, the standard treatment for mild hypoglycemia is administration of oral carbohydrate (glucose) followed by rechecking blood glucose levels after ~15 minutes and then repeating the process until normoglycemia is achieved. This approach often involves the consumption of excess, unwanted calories and leads to significant hyperglycemia, increasing time out of target glycemic range.

Many studies have demonstrated the successful use of MDG for the prevention and treatment of mild to moderate hypoglycemia in people with T1D. Four reports in the literature showed that MDG was very effective in the prevention and management of hypoglycemia during episodes of gastroenteritis and/or poor oral carbohydrate intake. In addition, this intervention decreased the frequency of hospital visits.8–10,12 Three published studies looked at the use of MDG for prevention of exercise-induced hypoglycemia. One demonstrated that MDG was more effective than reduction in basal insulin rate in preventing exercise- induced hypoglycemia and appeared to cause less post- exercise hyperglycemia compared to oral glucose administration.^13^ Another showed that glucose decline during exercise was diminished by pre-exercise MDG treatment.^14^ A third study found that MDG decreased exercise-associated hypoglycemia regardless of reduction in basal insulin rate.^15^ An assessment of MDG use in individuals with T1D who were fasting for Ramadan established that this intervention was effective for the prevention and treatment of hypoglycemia, allowing for safe and successful intervention in the setting of prolonged fasts.^16^ A letter to the editor describing the use of MDG in T1D children attending summer camp, where physical activity can cause more frequent episodes of hypoglycemia, showed that MDG corrected hypoglycemia quickly and effectively without complaints of nausea or vomiting from those treated. One study demonstrated that MDG may be effective for the treatment ethanol-induced hypoglycemia.^18^

Three studies describe the use of newer glucagon formulations in insulin-induced hypoglycemia. The first, a dose-response study utilizing a glucagon formulation stabilized by DMSO (the previous formulation had an aqueous base), showed that MDG derived from this new preparation was safe and effective with the optimal dose being 150 μg.^6^ Another study of this same formulation demonstrated that MDG was useful for the treatment of mild hypoglycemia.^19^ The third study utilized a next- generation glucagon analog, dasiglucagon. Study investigators found that this preparation, when used to formulate MDG, was safe and effective with respect to preventing insulin-induced hypoglycemia and brought blood glucose levels up much faster than oral glucose.^21^

Many prominent T1D leadership organizations find the available data on MDG use for hypoglycemia compelling. As a result, they have included detailed guidance regarding the use of MDG in their publications and on their websites. ISPAD’s published guidelines from 2009 and 2022 include detailed recommendations regarding the use of MDG for sick day management in children and adolescents with T1D to correct blood glucose and avoid hospitalization. Specifically, they recommend the utilization of MDG for children and adolescents with impending or achieved hypoglycemia and gastrointestinal illness/poor oral intake. Based on most MDG studies, they suggest using a dose of 20 μg for children ≤2 years of age with the addition of 10 μg per year of age for children ≥3 to 15 years old (maximum dose 150 μg). They recommend repeating MDG at twice the initial dose if glucose fails to rise sufficiently after 30 minutes.5,24 Similarly, the Pediatric Endocrine Society and Children with Diabetes include comparable guidelines on their public websites.^25,26^ In addition, countless academic institutions across the world include this guidance on their websites.

Despite an overwhelming body of literature demonstrating that MDG is extremely safe and effective for the treatment of mild to moderate hypoglycemia in T1D, glucagon is not yet FDA approved for use in this manner for this indication. Further, glucagon is not currently produced in a way that can be easily used to administer MDG. The original formulation of glucagon comes in a kit and requires aqueous reconstitution of a crystallized powder. The resulting solution is administered in one large (1 mg) dose for severe hypoglycemia, and if it is not used immediately, it requires refrigeration and must be discarded after 24 hours. This 1 mg kit can be reconstituted and divided up into insulin syringes (1 unit = 10 μg) to make six 150 μg MDG doses, but all unused portions must be discarded within 24 hours. Therefore, this is an expensive and impractical way to generate and use MDG. The newer formulations of glucagon, which do not require reconstitution and are stable at room temperature, are also meant to deliver a large dose of glucagon in the setting of severe hypoglycemia, and do not lend themselves to the creation of MDG aliquots. While it can be done, it is an unrealistic and costly approach.

## Conclusion

Hypoglycemia and fear of hypoglycemia can be impediments to the attainment of optimal glycemic control in T1D. Mini-dose glucagon is a safe and effective intervention for the prevention and treatment of mild to moderate hypoglycemia in individuals with T1D. It causes less hyperglycemia and avoids the ingestion of extra calories and the unwanted weight gain that can result from oral glucose administration. In addition, it decreases unnecessary hospital visits for mild to moderate hypoglycemia. A room temperature-stable, affordable glucagon formulation packaged as a multi-dose device is needed. The device should allow the user to adjust the MDG in increments of 10 μg from 20 μg (minimum dose) to 300 μg (maximum dose). In conjunction with a device, FDA approval of MDG for the treatment of mild to moderate hypoglycemia must be pursued. This advance in the field of T1D will safely and effectively address mild to moderate hypoglycemia to decrease the complications of both hypoglycemia and hyperglycemia.
